# Diseases of the Stomach and Intestines

**Published:** 1911-06

**Authors:** 


### BOOKS AND AUTHORS

**Diseases of the Stomach and Intestines.** By **Boardman Reed,** M.D., Consulting Gastroenterologist to the Pottenger Sanatorium, Monravia, Cal. Third edition. Octavo, pp. 1037. Illustrated. New York: E. B. Treat & Co. 1911. (Cloth, $5.00.)

The first part of this work deals with anatomic, physiologic, chemic and diagnostic data; the second with methods of examination; the third with methods of treatment; and the fourth, occupying two-thirds of the space, is termed the gastrointestinal clinic and deals systematically with the individual diseases and symptom complexes. This space proportion as well as the title of clinic, well indicates the stress laid by the author on practical issues, though by no means to the exclusion of scientific considerations.

Among some of the topics considered, in addition to the ordinary subjects, are arteriosclerosis, lesions of the liver, heart, kidneys and blood in their relation to gastroenteric conditions, the surgery of the stomach and intestine, including a discussion of peritoneal adhesions, Meckel's diverticulum and the like. This list affords an idea of the completeness of the work.

We are pleased to note quotations from the studies of three of our Buffalo men. Dr. Reed's early life included patriotic service in the civil war. His professional reputation, made at Atlantic City, was increased in Philadelphia and he has added to it in Los Angeles. The illustrations—112 in all—serve to explain the text more fully. We take pleasure in commending his perfected book not only to the specialist but to the general practitioner. A. L. B.

